# Digital Accuracy of Closed-Tray Implant Impressions: Influence of Polyvinyl Siloxane Viscosity and Subgingival Posterior Implant Angulation

**DOI:** 10.3390/dj13090421

**Published:** 2025-09-12

**Authors:** Yousra Ahmed, Shereen Moselhy Abdul Hameed, Zainab Refaey El Sharkawy, Faris A. Alshahrani, Tarek AbdAllah Mahmoud, Inas M. Mohamed, Noha Taymour

**Affiliations:** 1 Department of Prosthetic Dentistry, Removable Prosthodontic Division, Faculty of Dentistry, King Salman International University, El Tur 45615, Egypt; yousra.elsayed@ksiu.edu.eg; 2 Department of Crowns and Bridges, Faculty of Dental Medicine for Girls, Al Azhar University, Cairo 11765, Egypt; sherine.moselhy@gmail.com (S.M.A.H.); zainabelsharkawy.26@azhar.edu.eg (Z.R.E.S.); 3 Department of Substitutive Dental Sciences, College of Dentistry, Imam Abdulrahman Bin Faisal University, P.O. Box 1982, Dammam 31441, Saudi Arabia; 4 Department of Removable Prosthodontic, Faculty of Oral and Dental Medicine, Modern University for Technology and Information (MTI), Cairo 11585, Egypt; tarek.mahmoud@dnt.mti.edu.eg; 5 Lecturer of Dental Biomaterials Department, Faculty of Dental Medicine for Girls, Al-Azhar University, Cairo 11765, Egypt; enasalsherbiny@gmail.com

**Keywords:** subgingival implant, implant angulation, closed-tray impression, implant cast accuracy, polyvinyl siloxane

## Abstract

**Objectives:** To assess the impact of PVS impression material viscosity and implant angulation on the three-dimensional accuracy of implant casts in a partially edentulous situation using the closed-tray technique. **Materials and Methods**: Three epoxy resin mandibular partially edentulous models (Kennedy Class I) were fabricated, each with four implant analogues placed at teeth positions 35, 37, 45, and 47. The anterior analogues were positioned parallel (0), while the posterior analogues were placed at different angulations: Group 1, 30° mesiodistal; Group 2, 20° mesiodistal; Group 3, 20° buccolingual. All analogues were placed 2 mm subgingivally. Closed-tray impressions (n = 8 per subgroup) were made using either heavy + light body PVS or monophase PVS. Resulting stone casts were scanned, and STL files were processed and analyzed using reverse engineering software (Geomagic Control X). Three-dimensional deviations (root mean square, RMS) between reference and test models were calculated by superimposition and best-fit algorithm. **Results:** With monophase PVS, implant angulation significantly influenced cast accuracy (*p* < 0.001). The 30° MD group exhibited the highest deviation (96 ± 7 µm), followed by the 20° BL group (81 ± 6 µm), then the 20° MD group (75 ± 6 µm). In contrast, no statistically significant difference in accuracy was observed among angulation groups when using heavy + light body PVS (77 ± 3 µm, 82 ± 13 µm, and 79 ± 8 µm for 30° MD, 20° BL, and 20° MD, respectively; *p* = 0.550). **Conclusions:** Both monophase and heavy + light body PVS impression materials produced clinically acceptable accuracy for closed-tray implant impressions. However, the heavy + light body PVS demonstrated greater consistency across various implant angulations and is recommended for multiple angulated subgingival posterior implants when using the closed-tray technique.

## 1. Introduction

The restoration of partial and complete edentulism with osseointegrated implants has emerged as the preferred standard of dental care in modern prosthetic dentistry [1,2]. Unlike natural teeth, dental implants lack physiological mobility, making the achievement of a passive fit in implant-supported prostheses essential for long-term success [3]. Inadequate fit can induce stresses within the prosthetic superstructure, implants, and surrounding peri-implant tissues, leading to biological and mechanical complications [4,5]. Biologic problems may include peri-implant mucositis, peri-implantitis, and eventual implant failure [6], while mechanical complications often manifest as screw loosening or fracture, stripping of internal threads, or fracture of the implant or prosthetic framework components [7,8]. The dimensional accuracy of the working cast is fundamental to ensuring passive fit; thus, the precise three-dimensional transfer of implants, residual teeth, and adjacent anatomical structures during the impression procedure is a critical initial step [9].

Although intraoral scanners are increasingly used in clinical practice, conventional impression materials remain widely applied in daily dentistry. It is important to note that these materials may exhibit dimensional changes during the impression and model fabrication process [10]. For instance, impressions are typically taken at intraoral temperature (~37 °C) and poured at laboratory temperature (23–25 °C), which can result in slight contraction. Moreover, polyether impression materials are characterized by low water absorption, whereas polyvinyl siloxane (PVS) silicones release hydrogen as a byproduct of polymerization, both of which can influence material shrinkage and dimensional stability [11].

The accuracy of implant impressions is multifactorial, including the type of impression material and technique, splinted impression copings, impression level (implant or abutment), implant angulation, and depth [12,13]. Numerous impression materials and techniques have emerged to optimize the definitive casts accuracy, which is vital for fabricating passively fitting prostheses. Among the most widely utilized methods are the indirect (transfer or closed-tray) and direct (pickup or open-tray) techniques [14]. The selection of impression material is also pivotal for achieving dimensional accuracy. Although no material is flawless, polyether (PE) and polyvinyl siloxane (PVS) are regarded as the materials of choice in implant prosthodontics [15]. Monophase polyether is especially effective for complete edentulism and multiple-implant scenarios due to its dimensional stability, rigidity, as well as hydrophilicity [16]. However, polyether’s limitations including low tear strength, potential for allergic reactions, and a relatively short working time, can pose challenges. Its high stiffness after setting may also complicate impression removal, particularly in areas with undercuts [17].

Conversely, vinyl polysiloxane (PVS) offers superior elastic recovery [18] and is often preferred in cases involving non-parallel or internally connected implants, where minimizing strain between the impression material and copings is crucial to prevent permanent deformation [16]. PVS’s favorable elastic properties facilitate easier removal of the set impression while maintaining high dimensional accuracy [19]. Since its introduction in the 1970s, addition silicone, commonly known as PVS, has demonstrated improved dimensional stability and wettability compared to condensation silicones, owing to its unique chemical structure [20]. Accordingly, the present study aims to compare the accuracy of implant impressions obtained using two polyvinyl siloxane (PVS) viscosities: monophase and a combination of heavy and light body.

Implants are frequently placed subgingivally to address esthetic concerns, anatomical variations, or soft tissue thickness. This positioning necessitates deeper placement of impression copings within the gingival tissue, reducing the supragingival portion available for contact with impression material [21]. Such a reduction in contact surface may compromise impression accuracy [22]. Therefore, this study investigates the accuracy of different PVS viscosities under conditions of deep subgingival implant placement.

While numerous studies have explored the effects of implant angulation and impression technique on impression accuracy in partially edentulous cases [21,23], data remain limited regarding the combined influence of impression material viscosity and implant angulation, particularly in the mesio-distal and bucco-lingual directions, on definitive cast accuracy. Furthermore, the interplay of these variables under deep subgingival placement has not been thoroughly investigated. This in vitro study was therefore designed to evaluate the effects of impression material viscosity and implant angulation (mesio-distal and bucco-lingual) on the dimensional accuracy of implant casts in a partially edentulous model with subgingivally positioned implants. The null hypotheses were: (H_01_) impression material viscosity does not significantly affect the accuracy of digital implant casts; and (H_02_) implant angulation in the mesio-distal and bucco-lingual directions does not significantly influence the accuracy of casts when implants are positioned below the gingival margin.

## 2. Materials and Methods

### 2.1. Ethical Approval

Ethical approval for the study was obtained from the Research Ethics Committee of the Faculty of Dental Medicine for Girls, Al-Azhar University, in July 2025, Code: REC-PD-25-15.

### 2.2. Sample Size Calculation

Eight impressions were made per subgroup, based on a prior study [24]. The minimum sample size was six per group (effect size 1.49, α = 0.05, power = 80%), increased to eight to account for a 20% dropout rate. Sample size calculations were performed using G*Power 3.1.9.7 [25,26].

### 2.3. Master Model Preparation

**Figure 1:**
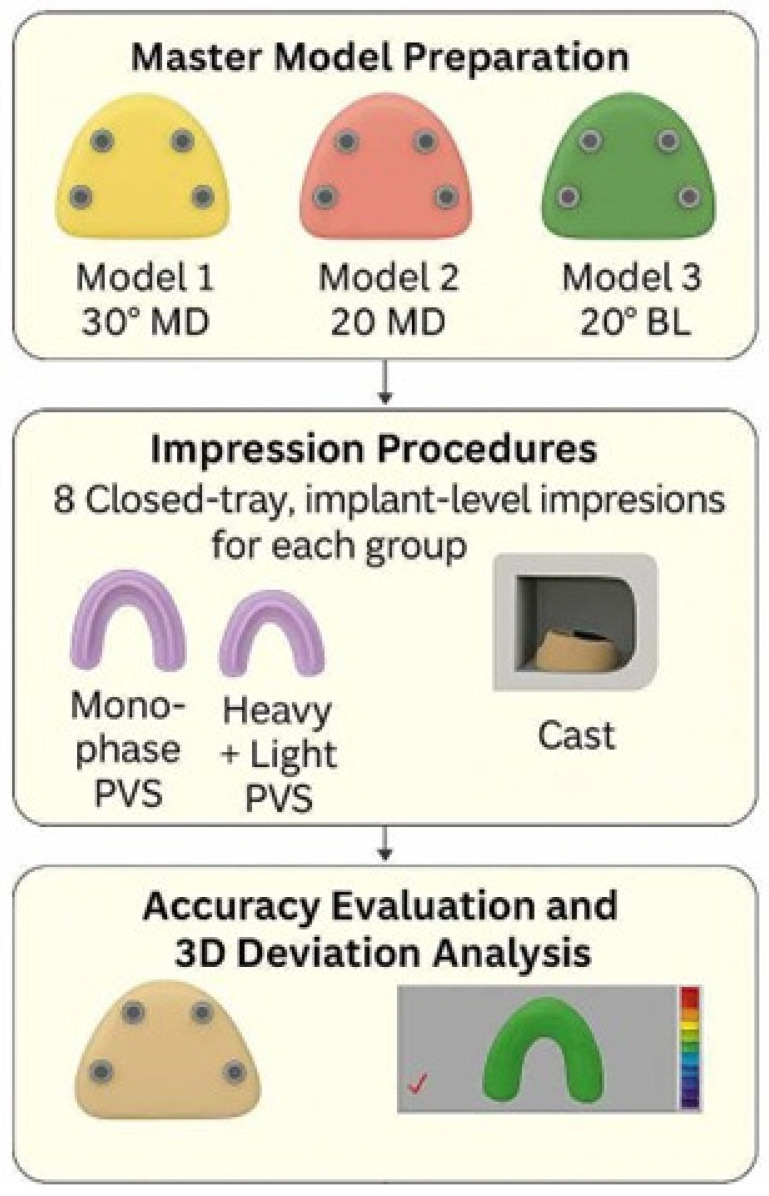
Study design flowchart.

A commercially available Kennedy Class I partially edentulous mandibular model was duplicated three times using solvent-free transparent epoxy resin to create three master models. Each resin model was precisely drilled using a 5-axis CNC machine to accommodate four internal connection dental implant analogues (Implant Direct, Sybron International), positioned at teeth number 35, 37, 45, and 47 according to FDI notation. In all models, the two anterior analogues were placed parallel (0°), while the two posterior analogues were positioned at different angulations: (Figure 1).

- Model 1: 30° mesiodistal angulation (tilted distally, Group 1: 30° MD).
- Model 2: 20° mesiodistal angulation (tilted distally, Group 2: 20° MD).
- Model 3: 20° buccolingual angulation (converging lingually, Group 3: 20° BL).

### 2.4. Custom Trays Fabrication

Custom trays were fabricated for closed-tray, implant-level impressions. For each master model, implant-level impression transfers were attached to analogues, and three layers of baseplate wax were adapted over and around the copings [27]. Two circular tissue stops (2 mm wide, 1 mm deep) were created to standardize tray placement. Using autopolymerizing resin, two 2-mm-thick perforated trays were made per model. Tray-fitting surfaces were coated with Universal Tray Adhesive and allowed to dry for two minutes before use. This process produced eight trays per model [22]. All study materials and equipment used are listed in Table 1.

The autopolymerizing resin was mixed at the manufacturer’s recommended 1:1 resin-to-monomer ratio to limit shrinkage and ensure proper polymerization. Trays were allowed to fully polymerize (15–20 min), then all of the trays were left for more 24 h before use, minimizing dimensional changes and ensuring accuracy [10].

### 2.5. Impression Procedures

Polyvinyl siloxane impressions were mixed using a mixing syringe to standardize the mixing process. As per standard procedure, the first part of the material was extruded and discarded to ensure the material was fully mixed in even quantities of base and catalyst. This step was taken to guarantee uniformity and accuracy in the impression material before application to the tray. All impressions were performed at 25 °C ± 2 °C. Copings were attached to analogues with a torque controller at 10 N/cm. Periapical radiographs confirmed seating of impression copings. Vinyl gloves were used to prevent retardation from latex interaction. Coping was secured with a hex tool. Heavy body PVS was loaded into the tray, while light body was injected around the copings. The tray was seated with light finger pressure onto the location marks and maintained during polymerization. A 1.5 kg metal block was placed on the tray to standardize pressure. After 4 min, the tray was removed with a sharp snap.

Monophase PVS Technique: Copings were screwed onto the analogues first using a torque controller set to 10 N/cm. Regular viscosity monophase PVS was dispensed using an auto mix gun. The material was then applied both in the tray and around the copings. The tray was positioned and seated using finger pressure, and a 1.5 kg metal block was used to standardize pressure during the setting process. After 6 min, the tray was removed with a sharp snap. Impressions were inspected and repeated if defects, such as air voids or material separation, were detected. All components were verified for correct orientation and seating. The impression copings were removed from the cast, attached to the analogues, and reseated in the impression. The same operator secured the analogues to the copings at 10 N/cm to ensure consistent and accurate placement.

### 2.6. Cast Fabrication and Scanning

**Figure 2:**
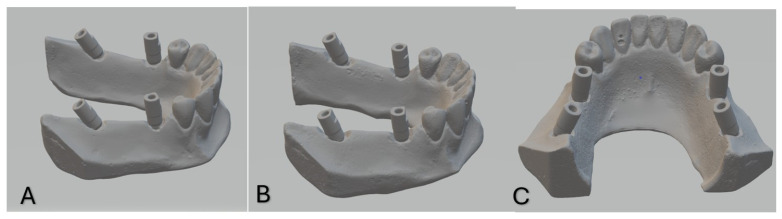
Reference scans of models 1, 2 and 3 ((**A**), (**B**) and (**C**), respectively).

Impressions were poured with type IV dental stone. The mixing ratio of gypsum to water for the type IV dental stone was 100 g of gypsum to 20 mL of water, as per the manufacturer’s guidelines, to ensure consistency and optimal flow. This procedure was performed by a single trained operator to ensure consistent mixing, pouring, and handling to minimize variability during the process. After 24 h, the casts were separated from impressions. PEEK scan bodies (Implant Direct, Sybron International) were attached to the analogues. All casts and master models were scanned using a laboratory scanner (Smart Optics, Activity 885, Germany) to produce STL files. Scan bodies were hand-tightened to a maximum of 15 N/cm, and the same scan bodies were transferred between models to eliminate variability. STL files of reference and test models were archived for analysis [28] (Figure 2).

### 2.7. Accuracy Evaluation and 3D Deviation Analysis

**Figure 3:**
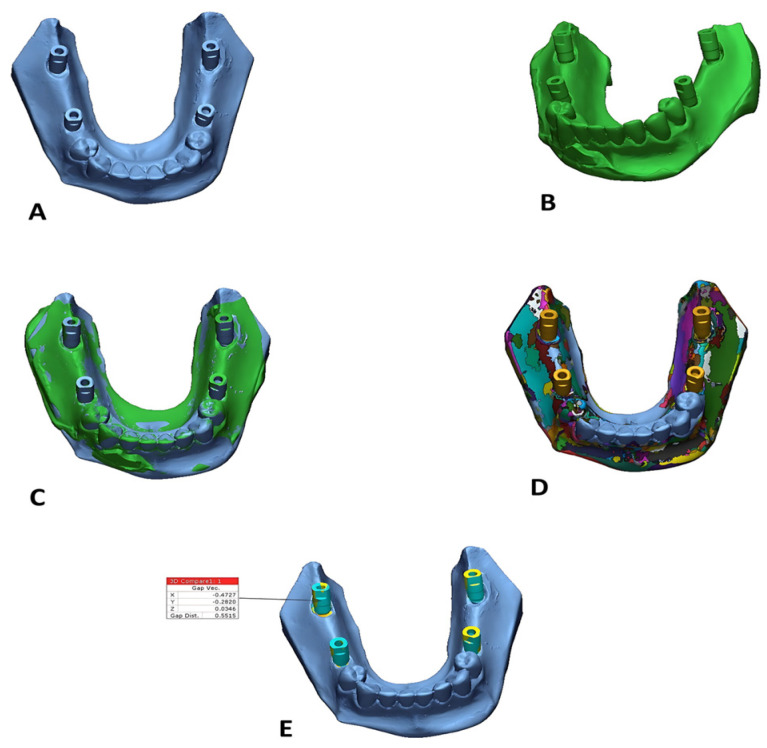
Superimposition of reference and test models: (**A**) reference model, (**B**) test model, (**C**) after the alignment of both reference and test models, (**D**) after the segmentation, (**E**) 3D comparison around the scan bodies only.

Digital datasets were evaluated utilizing industrial reverse engineering software (Geomagic Control X 2022, 3D Systems, Rock Hill, SC, USA). All STL files were examined for anomalies. The accuracy of each impression material was assessed by 3D deviation analysis [29] (Figure 3). The accuracy of each impression material was determined by comparing the STL files of the test models with those of their corresponding reference models. Each reference model was divided into two segments: the alveolar ridge and the teeth/abutments. For the three-dimensional superimposition and deviation analysis, only the teeth and abutments were utilized, applying an initial alignment followed by a best-fit algorithm. This segmentation ensured standardized comparison by excluding irrelevant areas. The superimposition software was directed to perform the best-fit alignment solely on the dentate regions, deliberately excluding the partially edentulous area where implants were placed. This strategy helped prevent distortion of alignment results and ensured a more accurate assessment of implant position discrepancies [16]. All procedures were performed according to standardized protocols to ensure reproducibility and accuracy.

### 2.8. Statistical Analysis

The numerical data were assessed for normality by evaluating their distribution and applying both the Kolmogorov–Smirnov and Shapiro–Wilk tests. All datasets demonstrated a normal (parametric) distribution. Results are reported as mean values with corresponding standard deviations (SD). A two-way analysis of variance (ANOVA) was conducted to evaluate the influence of impression material, implant angulation, and their interactions on the mean trueness, expressed as root mean square (RMS, in millimeters). When the ANOVA indicated significant differences, Bonferroni’s post hoc test was employed for pairwise comparisons. The threshold for statistical significance was set at *p* ≤ 0.05. All statistical analyses were performed using IBM SPSS Statistics for Windows, Version 23.0 (IBM Corp., Armonk, NY, USA).

## 3. Results

The two-way ANOVA analysis revealed that implant angulation, independent of the type of impression material, had a statistically significant impact on mean trueness (*p* = 0.032). In contrast, the viscosity of the impression material, regardless of angulation, did not show a statistically significant effect on mean trueness (*p* = 0.088). Notably, the interaction between angulation and impression material viscosity was statistically significant (*p* = 0.003), indicating that these factors are interdependent in influencing the accuracy of the implant impressions (Table 2).

Regardless of the impression material used, a statistically significant difference was observed between implant angulations (*p* = 0.032). Pairwise comparisons indicated that the 30° mesio-distal (MD) angulation group exhibited the greatest deviation, which was not significantly different from the 20° bucco-lingual (BL) group, but was significantly higher than the 20° MD group, which demonstrated the lowest deviation values. Conversely, when considering angulation, no statistically significant difference was found between the two impression materials (*p* = 0.088) (Table 3).

Comparison Between Angulations: When using the monophase PVS impression, a statistically significant difference was observed among the angulations (*p* < 0.001). Pairwise comparisons showed that the 30° mesio-distal (MD) group exhibited the highest mean deviation value (96 ± 7 µm), which was significantly greater than the 20° bucco-lingual (BL) group (81 ± 6 µm). The 20° MD group recorded the lowest mean deviation (75 ± 6 µm), which was also statistically significantly less than the other groups. In contrast, with the heavy and light body PVS impression, no statistically significant differences were found between the angulations (mean deviations of 77 ± 3 µm, 82 ± 13 µm, and 79 ± 8 µm for the 30° MD, 20° BL, and 20° MD groups, respectively) (*p* = 0.550).

**Figure 4:**
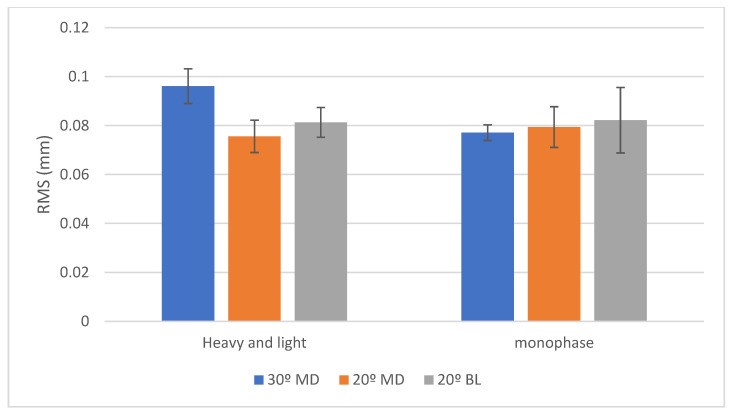
Bar chart showing RMS values (in mm) for trueness with two viscosities of silicone impression materials. Posterior implants were positioned at 30° MD (blue), 20° MD (orange), and 20° BL (gray) angles. Error bars represent standard deviation.

Comparison Between Impression Materials: At the 30° MD angulation, the monophase PVS impression yielded a significantly higher mean deviation (96 ± 7 µm) compared to the heavy and light body PVS combination (77 ± 3 µm) (*p* < 0.001). However, for the 20° MD and 20° BL angulations, no significant differences were identified between the two impression materials (*p* = 0.420 and *p* = 0.839, respectively). (Table 4) (Figure 4).

## 4. Discussion

This in vitro study investigated the influence of implant angulation and polyvinyl siloxane (PVS) impression material viscosity on the trueness of definitive implant casts using the closed-tray technique in a partially edentulous model with subgingivally placed implants. The closed-tray technique was selected in the present study due to its ease of use, reduced chairside time, and lower risk of distortion [30,31]. It has been reported that, particularly in cases involving angulated implants, the closed-tray technique may reduce dimensional distortion compared to the open-tray method which can introduce stress during tray removal and result in impression inaccuracies [30]. One of the primary approaches commonly employed for evaluating impression accuracy is the three-dimensional (3D) superimposition of standard tessellation language (STL) test files onto reference datasets using a best-fit algorithm based on the least squares method [13,20,21]. In this study, the 3D superimposition method using the best-fit algorithm was adopted to evaluate the dimensional accuracy of the impressions.

To replicate this clinically relevant scenario where insufficient bone volume or thick, soft tissue necessitates subgingival implant positioning, the implant analogues in the present study were placed 2 mm below the gingival margin [32]. Linkevicius et al. reported that the subgingival placement of implants significantly compromises the precision of impressions [33]. Additionally, it has been found that deeper implant placement diminishes the mechanical stability of the impression coping, thereby negatively affecting the accuracy of implant position transfer [34]. This study was conducted in vitro as the trueness of impression techniques is typically assessed by comparison with a high-precision reference dataset, which is challenging to obtain under in vivo conditions [35]. Epoxy resin reference models were utilized in this investigation due to their favorable mechanical properties, including an elastic modulus comparable to that of human cortical bone and greater dimensional stability than conventional dental stone models [22]. To generate reference digital datasets, a high-precision laboratory scanner was employed as it offers a precision of 6 µm and features laser triangulation technology, a white LED light source, wide measurement field, automatic 3D calibration, and high-speed fully automated scanning capability. According to the existing literature, laboratory reference scanners used in such studies typically provide a trueness range of 5–30 µm, which is considered acceptable for validating digital impression accuracy [36,37]. Pick-up impressions are conventionally performed using open trays; however, preparing and customizing the open tray can complicate the clinical procedure. Moreover, clinical limitations such as restricted inter arch space may hinder access to the pick-up coping’s retaining screw, rendering the open-tray technique impractical in certain scenarios. Conversely, transfer (closed-tray) copings are simpler to use and offer broader clinical applicability [38]. It has been reported that, particularly in cases involving angulated implants, the closed-tray technique may reduce dimensional distortion compared to the open-tray method which can introduce stress during tray removal and result in impression inaccuracies. Based on these considerations, the closed-tray technique was selected in the present study due to its ease of use, reduced chairside time, and lower risk of distortion [30].

The study revealed that heavy and light body PVS produced significantly lower RMS deviations compared to monophase PVS, particularly in 30 degrees angulated implant groups. Consequently, the first null hypothesis (H_01_), stating that impression material viscosity has no effect on impression accuracy, was rejected.

These findings coincide with those of Richi et al., who found that implant angulation, impression technique, and coping type significantly affect the accuracy of impressions, particularly in cases involving multiple angulated implants [39]. However, our results differ from those of Ghahremanloo et al. [40], who found monophase PVS to be more accurate than putty/light-body combinations, attributing this to the putty’s high filler content and limited tray space, which may restrict elastic recovery. Aidasani et al. [41] also reported higher precision with monophase polyether compared to vinyl polysiloxane and vinyl siloxanether, particularly in rotational discrepancies, although their study used the open-tray technique. The methodological differences, especially the use of the closed-tray technique in our study, likely account for these discrepancies. The rigid nature of polyether may minimize rotational error in open-tray techniques but could increase distortion during impression removal in closed-tray techniques, particularly at steep angulations.

Regarding implant angulation, our results indicated that that increased implant angulation, particularly at 30° mesiodistal angle (MD), was associated with higher deviation in trueness, especially when monophase PVS was used. Thus, the second null hypothesis (H_02_), that implant angulation does not affect impression accuracy, was also rejected.

It is worth noting that both impression viscosities produced accuracy results within the clinically acceptable threshold of 100 µm linear deviation, which is generally considered sufficient to ensure adequate passive fit and long-term prosthetic success [9,16,42]. This threshold applies even for implants placed 2 mm subgingivally and at angulations up to 30° distally or 20° lingually [43,44]. Nevertheless, heavy + light body PVS is recommended in such clinical scenarios due to its superior accuracy and lower three-dimensional deviation values.

A previous study reported that angulations up to 15° had no substantial effect on impression accuracy when using monophase PVS [16]. These findings are consistent with our results, which demonstrated comparable accuracy for impressions at moderate angulations when a monophase viscosity was used. Our findings also align with those of Parameshwari et al. [13] and Elshenawy et al. [45], who observed that higher implant angulations are associated with reduced impression accuracy, likely due to increased mechanical strain and deformation of the impression material. Filho et al. [46] similarly reported greater angular discrepancies with inclined implants, particularly when using monophase polyether. Sorrentino et al. [47] found that heavy- and light-body VPS outperformed monophase materials in capturing details of non-parallel implants, supporting our results.

The direction of implant angulation also plays a critical role in impression accuracy. Baldissara et al. noted that buccolingual divergence contributes significantly to impression distortion, particularly when using transfer copings [48]. While mesiodistal angulation primarily distorts the material along one axis, buccolingual angulation induces distortion in both buccolingual and mesiodistal planes, compounding the mechanical strain on the impression material. Additional distortion may occur during tray removal, especially in posterior regions, due to anteroposterior tilting. Our results corroborate these findings, demonstrating reduced accuracy in buccolingually angulated scenarios [42].

The interaction between implant angulation and impression material was further highlighted by Vojdani et al., who found that while impression material had minimal impact under parallel implant conditions, heavy and light body PVS performed significantly better in nonparallel configurations. This supports the use of dual-viscosity PVS in complex angulated implant cases due to its superior elastic recovery and flow properties [25].

Conrad et al. also agreed with our results, as they reported that implant angulation (5°, 10°, 15°), implant number, and impression technique did not significantly affect cast accuracy when using heavy and light-body PVS, suggesting that moderate angulations may not substantially compromise trueness with favorable elastomeric materials [38]. However, Shim et al. found that buccolingually angulated implants exhibited greater errors than parallel or mesiodistally angulated implants when using dual-viscosity PVS, although no significant difference was detected between parallel and mesiodistal groups. Variations in impression material brands, coping design, reference model configuration, and assessment methods (linear, angular, or 3D trueness) may explain these discrepancies [42]. Although angulated implants are often used to overcome anatomical constraints, our findings suggest that impression accuracy can be affected under these conditions, particularly with monophase PVS. Alternatively, in selected clinical scenarios like full-arch rehabilitations or fixed partial dentures, the use of prosthetic cantilevers may reduce the need for multiple angulated implants while still achieving satisfactory function and aesthetics [49].

The use of Light and Heavy Body PVS materials in combination has been shown to improve the accuracy of impressions compared to Monophase PVS. The greater filler content in Heavy Body material helps reduce shrinkage after polymerization, leading to more stable dimensional properties. This is because the denser structure of Heavy Body material, due to the higher filler content, undergoes less dimensional change during setting. In contrast, Monophase PVS, with lower filler content, tends to shrink more during polymerization, which could impact the final accuracy of the impression.

Light and Heavy Body PVS materials improve accuracy due to the higher filler content in Heavy Body, which reduces shrinkage after polymerization. But the little filler content in the light body makes it more elastic upon removal from undercut created by angled implants. However, materials with higher hardness and greater filler content (like medium body) may experience lower elastic recovery, leading to potential distortion during tray removal [47].

Both impression material viscosities yielded trueness values within clinically acceptable limits of less than 100 (µm) [40]. However, the greater accuracy observed with heavy and light body PVS across all angulation groups suggests its suitability for complex clinical scenarios involving angulated, subgingivally placed implants. The results highlight the importance of matching impression material properties to the specific clinical situation to optimize prosthesis fit and longevity.

### Limitations and Recommendations

This study has some limitations that should be considered. First, it was conducted under in vitro laboratory conditions, which although standardized do not fully replicate the intraoral environment where saliva, soft tissue resilience, temperature changes, and patient movement can affect impression accuracy. In addition, the study compared only two PVS impression techniques with a single implant system and laboratory scanner. Other impression materials, implant systems, or digital intraoral scanners were not evaluated, which may limit the generalizability of the findings. Finally, the analysis focused on immediate trueness without assessing long-term dimensional stability or clinical performance.

Future studies are recommended to validate these findings in vivo under real oral conditions, to incorporate a wider range of impression materials and scanning technologies, and to examine the long-term stability of impressions and their influence on prosthetic fit. Expanding sample sizes, conducting multicenter investigations, and including patient-centered outcomes such as time efficiency and comfort would further strengthen the clinical relevance of future research

## 5. Conclusions

- Within the limitations of this in vitro study, both monophase and heavy + light body polyvinyl siloxane (PVS) impressions demonstrated clinically acceptable accuracy.
- The combination of heavy + light body PVS impressions provided enhanced accuracy compared to monophase impressions.
- The superiority of the heavy + light body PVS was most evident in cases with multiple angulated implants, particularly at a 30° distal angulation in sub-gingival positions, when using the closed-tray technique.
- These findings suggest that impression material selection should be carefully considered in challenging clinical scenarios involving angulated implants to optimize accuracy and prosthetic outcomes.

## Figures and Tables

**Table 1:** Materials and equipment used in the study.

Material/Equipment	Manufacturer (Country)	Model/Reference Number
Transparent epoxy resin	CMB International, Egypt	BN1443120720 E667 G
Autopolymerizing resin	Acrostone Dental Manufacturer, Egypt	
Universal Tray Adhesive	Zhermack, Badia Polesine, Italy	C700025
PVS (Heavy + Light Body)—Elite HD+	Zhermack, Badia Polesine, Italy	C202032 C203040
PVS (Monophase)—Hydrorise Monophase	Zhermack, Badia Polesine, Italy	C207007
Mixing syringe & dispensing gun	DENTP, China	
Type IV dental stone—Fujirock EP	GC Corp., Tokyo, Japan	890222
PEEK scan adapters	Implant Direct, Sybron International, USA	#8035-09
Internal connection implant analogues	Implant Direct, Sybron International, USA	Legacy 1™ Internal Analogue. #8035-06
Impression coping	Implant Direct, Sybron International, USA	Closed tray, implant level transfer #8035-05
5-axis CNC machine	YCM Fanuc, Yeong Chin Machinery Ltd., Taiwan	YCM-Fanuc 5X
Laboratory scanner—Activity 885	Smart Optics, Germany	Activity 885
Reverse engineering software	3D Systems, USA	Geomagic Control X (2022)

™ (Trademark symbol), # (Catalog or Part Number indicator).

**Table 2:** Two-way mixed model ANOVA results for the effect of different variables on mean trueness (RMS in mm).

Source of Variation	Type III Sum of Squares	df	Mean Square	*F*-Value	*p*-Value	Effect Size *(Partial Eta Squared)*	95% CI for η^2^
Angulation	0.001	2	0.0005	3.864	0.032 *	0.205	0.02–0.39
Impression material	0.0002	1	0.0002	3.115	0.088	0.094	0.00–0.27
Angulation × Impression material interaction	0.001	2	0.0005	7.12	0.003 *	0.322	0.08–0.49

df: degrees of freedom = (n − 1), *: Significant at *p* ≤ 0.05.

**Table 3:** The mean, standard deviation (SD) values and results of two-way ANOVA test for main effects of the two variables on trueness (RMS in mm).

Variables		Mean	SD	*p*-Value	*Effect Size (Partial Eta Squared)*	95% CI for η^2^
Angulation	30° MD	0.0866 ^A^	0.0112	0.032 *	0.205	
	20° MD	0.0775 ^B^	0.0074			0.02–0.39
	20° BL	0.0817 ^AB^	0.0099			
Impression material	monophase	0.0843	0.0108	0.088	0.094	0.00–0.27
	Heavy + light	0.0796	0.009			

*: Significant at *p* ≤ 0.05, Different superscripts indicate statistically significant differences. Same letter = no significant difference (*p* > 0.05). Different letters = significant difference (*p* ≤ 0.05). Combination (AB) = overlaps with both groups → not significantly different from either A or B.

**Table 4:** The mean, standard deviation (SD), 95% confidence intervals (CI), and results of two-way ANOVA test for comparison between trueness (RMS in mm) with different interactions of variables.

Impression Material	30° MD		20° MD		20° BL		*p*-Value	Effect Size *(Partial Eta Squared)*	95% CI for η^2^
	Mean	SD	Mean	SD	Mean	SD			
Monophase	0.0961 ^A^	0.0071	0.0756 ^C^	0.0066	0.0813 ^B^	0.0061	<0.001 *	0.409	0.19–0.55
Heavy + light	0.0771	0.0032	0.0794	0.0083	0.0822	0.0134	0.550	0.039	0.00–0.17
*p*-value	<0.001 *		0.420		0.839				
Effect size *(Partial eta squared)*	0.357		0.022		0.001				
95% CI for η^2^	0.11–0.49		0.00–0.15		0.00–0.09				

*: Significant at *p* ≤ 0.05, Different superscripts in the same row indicate statistically significant difference between angulations. 30° MD (A) is significantly higher than both 20° MD (C) and 20° BL (B). 20° MD (C) is the lowest and significantly different from the others. 20° BL (B) is significantly different from both A and C.

## Data Availability

Available through the following link: https://figshare.com/s/9d9bcd9a01dec91a9923, accessed on 1 August 2025.
